# Are the Newer Carbapenems of Any Value against Tuberculosis

**DOI:** 10.3390/antibiotics11081070

**Published:** 2022-08-07

**Authors:** Ximena Gonzalo, Francis Drobniewski

**Affiliations:** Department of Infectious Diseases, Faculty of Medicine, Imperial College, London W12 0NN, UK

**Keywords:** carbapenems, microdilution, antibiotic resistance, meropenem, faropenem, tebopenem, ertapenem

## Abstract

Our aim was to assess whether newer carbapenems with a better administration profile than meropenem (ertapenem, faropenem and tebipenem) were more effective against *Mycobacterium tuberculosis* including M/XDRTB and determine if there was a synergistic/antagonistic effect with amoxicillin or clavulanate (inhibitor of beta-lactamases that MTB possesses) in vitro. Whilst meropenem is given three times a day intravenously, ertapenem, though given parenterally, is given once a day, faropenem and tebipenem are given orally. Eighty-two clinical drug-sensitive and -resistant MTB strains and a laboratory strain, H37Rv, were assessed by a microdilution methodology against ertapenem, faropenem, tebipenem and meropenem with and without amoxicillin or clavulanic acid. Ertapenem showed a limited activity. The addition of amoxicillin and clavulanate did not translate into significant improvements in susceptibility. Sixty-two isolates (75.6%) exhibited susceptibility to faropenem; the addition of amoxicillin and clavulanate further reduced the MIC in some isolates. Faropenem showed a limited activity (MIC of 8 mg/L or lower) in 21 strains completely resistant to meropenem (MIC of 16 mg/L or higher). Fifteen of the meropenem-resistant strains were susceptible to tebipenem. Carbapenems’ activity has been reported extensively. However, there remains uncertainty as to which of them is most active against TB and what the testing methodology should be.

## 1. Introduction

Carbapenems were discovered to be active against non-tuberculous mycobacteria in the last decade of the 20th century [1]. They inhibit the L,D-transpeptidases present in *Mycobacterium* spp. [2,3,4].

For the treatment of tuberculosis (TB), however, the interest in these drugs grew later, with the advent of multidrug and extensively drug-resistant tuberculosis (M/XDRTB). *Mycobacterium tuberculosis* (MTB) possesses a class C beta-lactamase that can inactivate carbapenems. Since the early 2000s, reports of good in vitro and in vivo results have emerged [5,6,7]. Of all the drugs in this class, meropenem proved to be the most stable against the chromosomally encoded *blaC* beta-lactamase [8]. The addition of clavulanic acid improves carbapenem activity probably by inhibiting beta-lactamase [3,9,10,11,12,13].

Co-amoxiclav has been included in the WHO Group C (former group V) antituberculous drugs for many years, despite the paucity of data to support its use [14,15,16]. Clavulanate (the beta-lactamase inhibitor in co-amoxiclav) can be administered orally. However, it is not available in the UK alone, but only in combination with antibiotics such as amoxicillin [13].

Concerns about pharmacological antagonism between amoxicillin and meropenem by means of completion for the binding sites have arisen. However, the addition of amoxicillin to meropenem and clavulanate shows a synergistic effect against *M. tuberculosis* strains at concentrations easily achievable in vivo [17].

Meropenem is given three times a day intravenously, and its use could drive emergence of resistance in the gut microbiota. Ertapenem, even though still requiring parenteral administration, is given once a day, while faropenem and tebipenem are given orally [13,18,19,20].

Faropenem is stable against the *blaC* enzyme present in MTB, which means that the drug is hydrolysed even if there is resistance to clavulanic acid [13,21]. When tested in an ex vivo model of TB using a laboratory strains H37Rv (but no clinical strains), faropenem manages to successfully kill MTB [22].

In one study, tebipenem showed a good activity in vitro against clinical isolates, including M/XDRTB [23].

Ertapenem has also been shown to be active in vitro. However, testing is challenging, since it degrades quickly at 37 °C [24,25].

The aim of this work was to assess the activity of carbapenems with a better administration profile against clinical strains of *M. tuberculosis*, including M/XDRTB.

## 2. Results

Ninety-three clinical strains and a laboratory strain, H37Rv, were assessed by a microdilution methodology against ertapenem, faropenem, tebipenem and meropenem and their combination with amoxicillin and clavulanic acid using a microtiter plate format. A picture of a plate ready to be read can be found in Supplementary Figure S1.

A readable susceptibility profile was obtained for 82 strains (87.2%). Eleven strains failed to grow in microtiter plates. A full set of results for drug-susceptible and drug-resistant strains can be found in Table 1 and Table 2, with the MIC_50_ and MIC_90_ values given in Table 3 and Table 4 for each group.

The MIC_50_ values for the faropenem−clavulanate combination with and without amoxicillin were 2 mg/L, while the MIC90 values were 32 mg/L. Sixty-two isolates showed an MIC of 8 mg/L or less, falling into the susceptibility category, and 62 isolates were susceptible to faropenem, clavulanate and amoxicillin.

The ertapenem−clavulanate combination showed an MIC_50_ of 32 mg/L that did not change with the addition of amoxicillin. The corresponding MIC_90_ was 64 mg/L. Twelve strains had an MIC of 4 mg/L or less, which is the cut-off based on PK/PD models for the current dose of 1 g once per day. If the dose is changed to 2 g twice a day, the cut-off is 16 mg/L in which case 34 strains is considered susceptible. With the addition of amoxicillin, 14 out of 82 strains show an MIC of 4 mg/L or less, and 34 strains presented an MIC of 16 mg/L or less.

The meropenem−clavulanate combination showed an MIC_50_ of 8 mg/L and decreased to 4 mg/L after amoxicillin was added. Its MIC_90_ was 64 mg/L. Forty-six strains had an MIC of 8 mg/L or less, which is the susceptibility cut-off for Gram-negative microorganisms. With the addition of amoxicillin, 57 strains were susceptible having MICs of 8 mg/L or less. A decrease in at least two-fold dilution is expected when synergy is present. A rise in MIC suggests antagonism, and no changes or reduction in less than two-fold dilution indicate an additive effect [17,26,27].

The tebipenem−clavulanate combination had an MIC_50_ of 2 mg/L that decreased to 1 mg/L after the addition of amoxicillin and its MIC_90_ was 64 mg/L. Sixty strains showed an MIC of 8 mg/L or less, which would be considered susceptible. This number grew to 64 when adding amoxicillin.

Of the 52 susceptible strains tested, 40 strains had a faropenem MIC of 8 mg/L or less. The addition of clavulanate increased the number to 41. For ertapenem, only H37RV had an MIC of 0.5 mg/L or less [28]. Twenty-eight isolates were susceptible to meropenem, and 35 isolates were susceptible to the meropenem−clavulanate combination. Forty strains had a tebipenem MIC of 8 mg/L or less. This number did not change with the addition of clavulanate.

Of the 27 MDRTB isolates (nine were XDRTB), 19 had a faropenem MIC of 8 mg/L or less, and the number increased to 21 after the addition of clavulanate. Three were susceptible to ertapenem using the EUCAST cut-off of 0.5 mg/L. Fifteen isolates were susceptible to meropenem, and 19 isolates were susceptible to the meropenem−clavulanate combination. Seventeen isolates had a tebipenem MIC of 8 mg/L or less, and the number was increased to 21 after the addition of clavulanate.

## 3. Discussion

Ertapenem showed limited activity with only a few isolates, demonstrating susceptibility. This lack of activity is potentially an artefact associated with the reported phenomenon of ertapenem degradation in vitro [25]. Given the slow replication of *M. tuberculosis*, this leads to a challenging situation in testing where the antibiotic possibly degrades before killing or inhibiting bacterial growth. Some authors have suggested the daily addition of antibiotics to the experimental setup [29], but this will hamper the evaluation of the dose tested and increase the risk of contamination as well as posing a repeated risk for the operator when working with M/XDRTB. The addition of the amoxicillin−clavulanate combination did not translate into significant improvements in susceptibility. Although ertapenem has been reported as useful in the treatment of TB, as part of combination therapy, its role remains unclear [7,24]. Previous animal studies reported an ertapenem MIC of 4 mg/L [7,13].

Faropenem is thermo-stable at 37 °C [30]. Sixty-two out of 82 isolates (75.6%) exhibited different degrees of susceptibility to faropenem, and the addition of amoxicillin and clavulanate further reduced the MIC in some isolates. This is in line with previous experiments with other carbapenems, in particular meropenem [17]. The current breakpoint for Gram-positive bacteria is 2 mg/L, which matches the MIC50 found in this study. The MIC for Gram-negative microorganisms is higher, i.e., 8 mg/L, which means that these antibiotic concentrations can be achieved in vivo [13,28].

Faropenem did show some limited activity (MIC of 8 mg/L or lower) in 21 strains completely resistant to meropenem (MIC of 16 mg/L or higher). Fifteen of the meropenem-resistant strains were susceptible to tebipenem. However, of the 52 isolates fully susceptible to first-line antituberculous drugs, 20 were resistant to the faropenem used, while 12 were resistant to tebipenem, indicating a role more confined to drug-resistant isolates. An additional hurdle for their clinical use is associated with the fact that routine susceptibility testing is not readily available.

Further information regarding the concentration of faropenem in blood and lung tissue is needed, as the MICs found in the current study were close to the cut-off value and there needs to be more certainty if those levels can be achieved with current dosing regimens [13].

In the last 20 years, carbapenems’ activity against mycobacteria has been reported extensively [13,31]. However, there is still a lack of certainty regarding which of them is the best against TB and what the best testing methodology is, as multiple methods with conflicting results have been reported [13,17,32,33]. In an animal model, in which Swiss mice were infected with MTB H37Rv, by comparing the results of the control group, the combination of a carbapenem and clavulanic acid improved survival in the treated group, but it did not stop the growth of the microorganism overall. The size of the spleen, the number of lung lesions and the colony-forming units in the lung were not different in treated and control mice [7]. In a more physiological hypoxic model emulating granuloma conditions, carbapenems have limited activity [34].

Mechanisms of resistance to carbapenems in mycobacteria remain poorly understood. MTB has efflux pumps that can potentially be at least partially implicated in resistance [35]. Changes in sulfolipids can increase impermeability, as has been observed for *M. bovis* BCG with ampicillin [36]. Additionally, a study in 2017 found that a mutation in a non-annotated protein confers resistance to the carbapenems meropenem and biapenem [37]. Lucic et al. [38,39] showed that resistance to faropenem is complex. Faropenem is orally active with a C-2 tetrahydrofuran (THF) ring, which is resistant to hydrolysis by some β-lactamases. They reported reactions of faropenem with carbapenem-hydrolysing β-lactamases, focusing on the class A serine β-lactamase KPC-2, the metallo β-lactamases (MBLs) VIM-2 (a subclass B1 MBL) and L1 (a B3 MBL). Kinetic studies showed that faropenem is a substrate for all three β-lactamases. Crystallographic analyses on faropenem-derived complexes revealed the opening of the β-lactam ring with the formation of an imine with KPC-2, VIM-2 and L1. In the cases of the KPC-2 and VIM-2 structures, the THF ring is opened to give an alkene, but with L1 the THF ring remains intact. Solution state studies, employing NMR, were performed on L1, KPC-2, VIM-2, VIM-1, NDM-1, OXA-23, OXA-10 and OXA-48. The solution results revealed, in all cases, the formation of imine products in which the THF ring is opened; the formation of a THF ring-closed imine product was only observed with VIM-1 and VIM-2. An enamine product with a closed THF ring was also observed in all cases at varying levels. Lucic et al. pointed out the potential for different outcomes in the reactions of penems with MBLs and SBLs and also demonstrated how crystal structures of β-lactamase substrate/inhibitor complexes do not always reflect reaction outcomes in solutions [38,39].

Clinical outcome evidence remains difficult to interpret, as therapy of MDR and XDR-TB involves combinations of several drugs. Currently, no well-powered control trial exists [31,40,41]. Due to the lack of clarity about effectiveness, higher costs associated with their use, the administration route and the potential emergence of resistance amongst gut microbiota, these drugs should be considered companion drugs rather than effective anti-TB agents [9,11,13,42,43,44,45].

Carbapenems showed modest in vitro activity using microdilution methods. The susceptibility is strain-specific and cannot be assumed a priori as it is not associated with M/XDRTB status.

Carbapenems cannot be considered active against *M. tuberculosis*, if the current EUCAST cut-off for Gram-positive microorganisms is followed. However, using PK/PD criteria, there are some activities and limited roles for meropenem, faropenem and tebipenem. The killing efficacy of the compounds tested was dose-dependent. Tebipenem was most efficient in killing MTB. The addition of clavulanate (2.5 mg/L) did not increase the killing efficacy of the antibiotics, except for meropenem.

More research is needed to clarify the roles of these antimicrobials in the treatment of *M. tuberculosis*. The recent introduction of novel inhibitors/carbapenems, such as ralebactam and varbobactam, could be explored.

## 4. Materials and Methods

Ninety-three clinical strains and a laboratory strain, H37Rv (Table 5), were tested against ertapenem, faropenem, tebipenem and meropenem and their combinations with amoxicillin and clavulanic acid using microdilution.

From frozen aliquots, the seed lot was generated by culturing it on Middlebrook 7H11 media. The plates were read twice a week. When growth was detected (more than 50 colony-forming units), colonies from Middlebrook 7H11 plates were suspended in 7H9 supplemented with OADC. Glass beads were added. The tube was shaken, until bacterial clumps were broken. The suspension was matched to a McFarland of 1 and left to rest for 20 min. One hundred microlitres were transferred to 11 mL of 7H9 supplemented with OADC and vortex for 20 s. Fifty microlitres of bacterial suspension were inoculated in each well (A, B, C, D, E and F) of a microtiter plate. The final volume per well was 0.1 mL.

The plate was sealed, double-bagged, placed in a plastic container and incubated at 37 °C and in a 5% CO_2_ atmosphere, and readings were performed weekly until 28 days. Plates were deemed ready for interpretation, when there was visible growth in the growth control wells (H11 and H12). Final concentrations of antibiotics per well can be found in Table 6. The minimal inhibitory concentration (MIC), which was the first well with no visible growth, was recorded for each strain.

## 5. Conclusions 

Carbapenems show limited activity when using PK/PD criteria. The killing efficacy of the compounds tested was dose-dependent. Tebipenem was most efficient in killing MTB.

## Figures and Tables

**Table 1:** MICs of carbapenems (mg/L) in association with clavulanic acid alone as well as clavulanic acid and amoxicillin for fully susceptible MTB strains. Clavulanic acid was used at a fixed concentration of 2.5 mg/L, and amoxicillin was used at a fixed concentration of 2 mg/L. Faro, faropenem; Clav, clavulanic acid; AMX, amoxicillin; Erta, ertapenem; MEM, meropenem; TEBI, tebipenem.

Strain Number	Faro + Clav	Faro + Clav + AMX	Erta + Clav	Erta + Clav + AMX	MEM + Clav	MEM + Clav + AMX	TEBI + Clav	TEBI + Clav + AMX
RT12000416	0.125	0.5	2	2	0.125	0.125	0.125	0.125
RT12000555	1	0.06	8	4	0.125	0.125	0.125	0.125
M08.14309	8	0.06	8	16	0.125	0.125	0.125	0.125
M08.14362	1	1	16	8	2	2	0.125	0.125
RT12000346	1	0.5	4	16	2	2	0.125	0.125
RT12000333	2	0.5	8	4	4	2	0.125	0.125
M08.14410	0.5	1	8	8	8	4	0.125	0.125
M08.14412	8	2	32	32	32	4	0.125	0.125
M08.14399	0.06	0.06	32	32	0.5	8	0.125	0.125
M08.14417	2	1	64	32	4	8	0.125	0.125
M08.14413	1	0.5	64	64	8	8	0.125	0.125
M08.14392	1	1	32	32	16	8	0.125	0.125
M08.14426	0.06	0.06	8	8	4	2	0.5	0.125
M08.14415	0.25	0.06	16	8	2	4	1	0.125
M08.14397	2	1	64	64	32	8	0.5	0.25
H112080012	2	2	16	16	8	1	0.125	0.5
H111900039	16	16	32	32	8	1	0.125	0.5
H37Rv	0.06	0.06	0.5	1	64	1	0.125	0.5
RT12000553	0.5	0.25	8	8	4	1	0.5	0.5
RT12000566	0.125	0.125	16	2	2	2	0.5	0.5
RT12000552	0.5	0.5	64	64	2	2	0.5	0.5
RT12000409	0.25	1	2	32	16	2	0.5	0.5
H111900041	8	16	64	64	4	0.5	2	0.5
M08.14408	4	1	64	64	16	8	16	0.5
3.013	2	0.25	4	4	0.5	0.5	0.5	1
RT12000338	0.06	0.06	16	0.5	4	2	1	1
RT12000326	0.06	1	16	16	0.5	4	1	1
RT12000324	2	0.5	8	16	8	8	1	1
M08.14422	8	8	64	64	64	8	1	1
M08.14400	2	8	64	64	32	32	1	1
2.292	1	0.5	8	16	0.5	0.5	2	1
RT12000328	4	1	8	8	4	4	1	2
5.177	16	4	32	32	32	16	4	2
M08.14361	32	4	64	64	8	8	5	2
M08.14432	2	1	64	32	32	16	4	4
M08.14440	4	4	64	64	32	32	4	4
M08.14437	16	16	64	64	64	32	4	4
M08.14363	4	8	16	16	8	4	8	4
H110860461	4	4	32	32	8	8	8	4
M08.14411	4	8	64	64	64	64	8	8
M08.14423	0.06	0.06	64	64	8	2	1	16
M08.14471	8	8	64	64	64	64	16	32
M08.14402	2	2	64	64	64	64	32	32
M08.14353	8	8	64	64	64	64	32	64
RT12000347	32	32	64	64	64	64	64	64
RT12000443	32	32	64	64	64	64	64	64
M08.14407	32	32	64	64	64	64	64	64
M08.14414	32	32	64	64	64	64	64	64
M08.14425	32	32	64	64	64	64	64	64
M08.14434	32	32	64	64	64	64	64	64
M08.14439	32	32	64	64	64	64	64	64
M08.14352	32	32	64	64	64	64	64	64

**Table 2:** MIC_50_, MIC_90_ and modal MIC values of carbapenems in association with clavulanic acid alone as well as clavulanic acid and amoxicillin for fully susceptible MTB strains. Clavulanic acid was used at a fixed concentration of 2.5 mg/L, and amoxicillin was used at a fixed concentration of 2 mg/L. Faro, faropenem; Clav, clavulanic acid; AMX, amoxicillin; Erta, ertapenem; MEM, meropenem; TEBI, tebipenem.

	Faro + Clav	Faro + Clav + AMX	Erta + Clav	Erta + Clav + AMX	MEM + Clav	MEM + Clav + AMX	TEBI + Clav	TEBI + Clav + AMX
MIC50	2	1	32	32	8	8	1	1
MIC90	32	32	64	64	64	64	64	64
Modal MIC	2	1	64	64	64	64	0.125	0.125

**Table 3:** MICs of carbapenems (mg/L) in association with clavulanic acid alone as well as clavulanic acid and amoxicillin for strains with resistance to one or more first- and second-line drugs. Clavulanic acid was used at a fixed concentration of 2.5 mg/L, and amoxicillin was used at a fixed concentration of 2 mg/L. Faro, faropenem; Clav, clavulanic acid; AMX, amoxicillin; Erta, ertapenem; MEM, meropenem; TEBI, tebipenem.

Strain Number	Faro + Clav	Faro + Clav + AMX	Erta + Clav	Erta + Clav + AMX	MEM + Clav	MEM + Clav + AMX	TEBI + Clav	TEBI + Clav + AMX
H111500010	0.06	0.06	0.5	1	0.125	0.125	0.125	0.125
M08.14304	0.06	0.06	4	0.125	2	0.5	0.125	0.125
H112080018	0.06	0.06	16	64	8	2	0.125	0.125
M08.14337	0.25	0.06	2	1	8	8	0.125	0.125
M08.14377	2	2	32	32	16	8	0.125	0.125
M08.14350	0.06	0.125	0.125	0.125	2	0.125	0.25	0.125
M08.14365	0.25	0.5	0.5	1	1	0.25	0.25	0.25
H111980010	4	2	16	16	1	1	4	0.25
M08.14303	2	2	16	8	4	1	0.5	0.5
M08.14354	1	2	16	16	8	4	1	0.5
M08.14310	1	4	32	16	16	8	2	1
M08.14493	16	16	32	32	2	0.5	8	1
H111040027	8	8	64	64	1	0.25	16	1
H111880072	32	32	64	64	2	2	8	2
H111620021	8	8	64	64	8	4	8	2
11.368	4	4	32	32	32	8	16	2
H111860011	2	3	4	8	1	1	4	4
M08.14358	0.06	0.06	32	64	8	2	4	4
H111740353	32	32	64	64	8	4	4	4
H112140033	1	0.5	0.125	0.125	16	32	4	4
H111540004	8	8	64	64	32	8	32	4
M08.14543	32	32	32	8	64	64	32	4
H111840003	0.25	0.06	16	4	4	4	8	8
H111620002	2	0.06	32	32	8	4	8	8
H112160033	1	1	8	16	16	16	16	16
M08.14306	16	8	64	64	16	16	16	16
M08.14366	16	16	32	32	32	32	32	32
M08.14486	8	8	32	32	64	32	32	32
H112990114	16	8	32	32	16	16	64	64
M08.14361	32	32	32	32	64	64	64	64

**Table 4:** MIC_50_, MIC_90_ and modal MIC values of carbapenems in association with clavulanic acid alone as well as clavulanic acid and amoxicillin for strains with resistance to one or more first- and second-line drugs. Clavulanic acid was used at a fixed concentration of 2.5 mg/L, and amoxicillin was used at a fixed concentration of 2 mg/L. Faro, faropenem; Clav, clavulanic acid; AMX, amoxicillin; Erta, ertapenem; MEM, meropenem; TEBI, tebipenem.

	Faro + Clav	Faro + Clav + AMX	Erta + Clav	Erta + Clav + AMX	MEM + Clav	MEM + Clav + AMX	TEBI + Clav	TEBI + Clav + AMX
MIC50	2	2.5	32	32	8	4	6	2
MIC90	16	16	64	64	32	32	32	16
Modal MIC	0.06	0.06	32	64	8	8	0.125	0.125

**Table 5:** Strains tested and their susceptibility profiles. S, fully susceptible; RIF, rifampicin-resistant; MDR, multi-drug resistant; POLYR, poly-resistant; XDR, extensively drug-resistant.

Strain Number	Phenotypical Resistance Profile	Strain Number	Phenotypical Resistance Profile
H37Rv	S	M08.14303	XDR
M08.14358	MDR	M08.14377	MDR
H111500010	MDR	H112080012	S
RT12000326	S	M08.14363	S
RT12000338	S	RT12000328	S
M08.14399	S	11.368	MDR
M08.14423	S	M08.14408	S
M08.14426	S	M08.14411	S
M08.14304	MDR	M08.14440	S
M08.14350	XDR	H110860461	S
H112080018	MDR	H111980010	POLYR
RT12000416	S	H111540004	MDR
RT12000566	S	H111620021	MDR
M08.14365	POLYR	M08.14412	S
RT12000409	S	M08.14422	S
M08.14415	S	H111040027	MDR
M08.14337	MDR	M08.14486	XDR
H111840003	RIF	M08.14471	S
RT12000552	S	H111900041	S
RT12000553	S	M08.14309	S
M08.14410	S	M08.14353	S
M08.14362	S	M08.14366	XDR
RT12000346	S	05.177	S
RT12000555	S	M08.14437	S
02.292	S	H111900039	S
M08.14392	S	H112990114	MDR
M08.14413	S	M08.14493	XDR
H112140033	MDR	M08.14306	XDR
H112160033	MDR	M08.14361	S
M08.14310	MDR	RT12000347	S
M08.14354	MDR	RT12000443	S
RT12000324	S	M08.14407	S
RT12000333	S	H111740353	MDR
H111620002	MDR	M08.14414	S
03.013	S	M08.14425	S
M08.14397	S	M08.14434	S
H111860011	MDR	M08.14439	S
M08.14400	S	H111880072	XDR
M08.14402	S	M08.14361	XDR
M08.14417	S	M08.14543	XDR
M08.14432	S	M08.14352	S

**Table 6:** Concentrations of ertapenem, faropenem, meropenem and tebipenem tested in combination with amoxicillin and clavulanate (in mg/L). F, faropenem; C, clavulanate; A, amoxicillin; M, meropenem; E, ertapenem; T, tebipenem.

	Concentration (mg/L)							
**F/C**	0.125 + 2.5	0.25 + 2.5	0.5 + 2.5	1 + 2.5	2 + 2.5	4 + 2.5	8 + 2.5	16 + 2.5
**F/C/A**	0.125 + 2.5 + 2	0.25 + 2.5 + 2	0.5 + 2.5 + 2	1 + 2.5 + 2	2 + 2.5 + 2	4 + 2.5 + 2	8 + 2.5 + 2	16 + 2.5 + 2
**E/C**	0.25 + 2.5	0.5 + 2.5	1 + 2.5	2 + 2.5	4 + 2.5	8 + 2.5	16 + 2.5	32 + 2.5
**E/C/A**	0.25 + 2.5 + 2	0.5 + 2.5 + 2	1 + 2.5 + 2	2 + 2.5 + 2	4 + 2.5 + 2	8 + 2.5 + 2	16 + 2.5 + 2	32 + 2.5 + 2
**M/C**	0.25 + 2.5	0.5 + 2.5	1 + 2.5	2 + 2.5	4 + 2.5	8 + 2.5	16 + 2.5	32 + 2.5
**M/C/A**	0.25 + 2.5 + 2	0.5 + 2.5 + 2	1 + 2.5 + 2	2 + 2.5 + 2	4 + 2.5 + 2	8 + 2.5 + 2	16 + 2.5 + 2	32 + 2.5 + 2
**T/C**	0.25 + 2.5	0.5 + 2.5	1 + 2.5	2 + 2.5	4 + 2.5	8 + 2.5	16 + 2.5	32 + 2.5
**T/C/A**	0.25 + 2.5 + 2	0.5 + 2.5 + 2	1 + 2.5 + 2	2 + 2.5 + 2	4 + 2.5 + 2	8 + 2.5 + 2	16 + 2.5 + 2	32 + 2.5 + 2

## Data Availability

All relevant data are included in the manuscript.

## Supplementary Materials

The following supporting information can be downloaded at: https://www.mdpi.com/article/10.3390/antibiotics11081070/s1. Figure S1: Microtiter plate ready to be read.
