# Supplementary figures and images for: Are the Newer Carbapenems of Any Value against Tuberculosis
Source: Antibiotics (Basel). 2022 Aug 7;11(8):1070. doi: 10.3390/antibiotics11081070 (PMC9404707; doi:10.3390/antibiotics11081070)

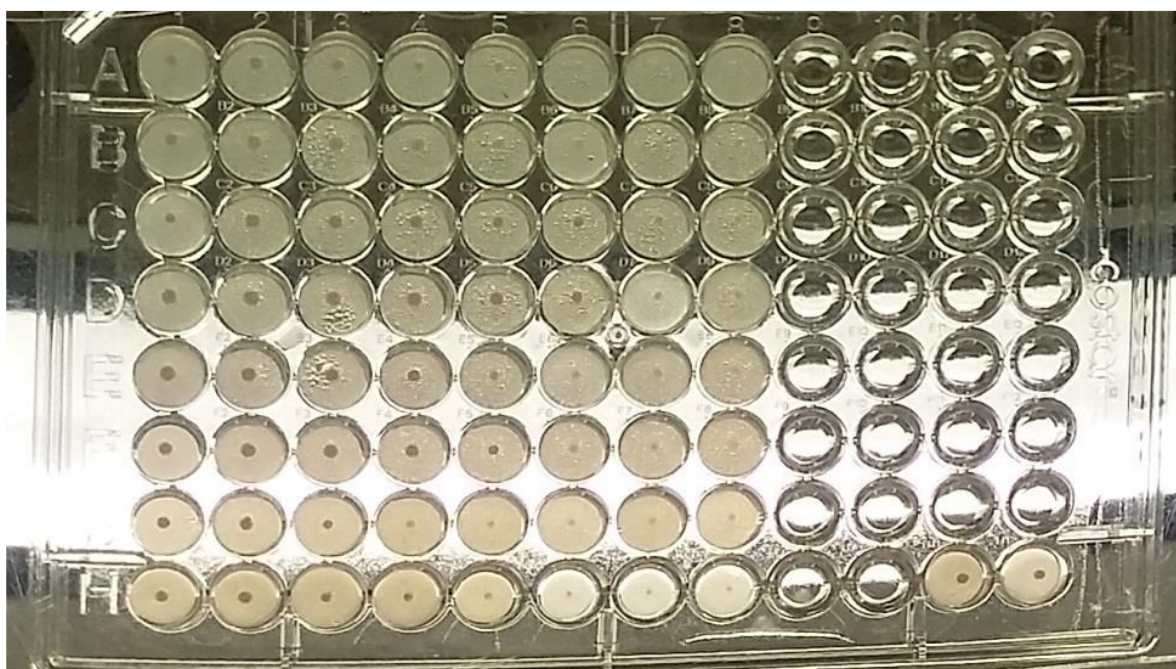

**Figure S1.** Microtiter plate ready to be read.

Supplement: Supplementary file 1 [file antibiotics-11-01070-s001.zip › antibiotics-1836247-supplementary.pdf]
